# Serum sodium as an adjunct marker of acute appendicitis severity: a retrospective cohort study

**DOI:** 10.1186/s12893-023-02224-y

**Published:** 2023-10-14

**Authors:** Bruno Messias, Isabella Cubas, Caio Oliveira, Flavia Hashimoto, Erica Mocchetti, Tania Ichinose, Jaques Waisberg, Marcelo A. F. Ribeiro Junior

**Affiliations:** 1 Department of Surgery, General Hospital of Carapicuiba, 95, Pedreira street, Carapicuiba, 06321-665 SP Brazil; 2 Medical School, São Camilo University Center, Nazare Avenue, São Paulo, 1501, 04263- 200 SP Brazil; 3 Department of Surgery, ABC Medical School, Lauro Gomes Avenue, Santo André, 2000, 09060-870 SP Brazil; 4 Critical Care and Acute Care Surgery, Sheikh Shakhbout Medical City- Mayo Clinic, P. O. Box 11001, Abu Dhabi, United Arab Emirates; 5 Catholic University of São Paulo, 290, Joubert Wey Street, Sorocaba, 18030-070 SP Brazil

**Keywords:** Acute appendicitis, Hyponatremia, Appendectomy, Complicated appendicitis

## Abstract

**Background:**

Early and accurate preoperative diagnosis of complicated appendicitis mandates the identification of new markers. The aim of this study is to determine whether preoperative serum sodium levels are useful for predicting the severity of acute appendicitis.

**Methods:**

We retrospectively analyzed 475 patients who underwent emergency appendectomies between January 2018 and February 2023 in a general hospital in Brazil. The patients were divided into 2 groups: complicated (n = 254) and uncomplicated (n = 221). Hyponatremia was defined as serum sodium levels < 136 mEq/L. The primary outcome was to evaluate if hyponatremia is associated with complicated appendicitis.

**Results:**

The patients had a median age of 22 years, and the median serum sodium level was 137 mEq/L in patients with complicated appendicitis and 139 mEq/L in uncomplicated appendicitis (*P* < 0.001). The analysis of the receiver operating characteristic curve used as the best cutoff value of serum sodium of 136 mEq/L with a sensitivity of 45.7%, specificity of 86.4%, positive predictive value of 79.5%, and negative predictive value of 58.1% for the diagnosis of complicated AA. Of the 254 patients with complicated appendicitis, 84 (33.1%) had serum sodium levels below 136 mEq/L, while only 12 (5.4%) patients with uncomplicated appendicitis had values ​​below this cutoff. Patients with hyponatremia were 5 times more likely to develop complicated appendicitis. (odds ratio: 5.35; 95% confidence interval: 3.39–8.45)

**Conclusions:**

Preoperative serum sodium levels may help in assessing the severity of acute appendicitis. Given their low cost and wide availability, sodium measurements could be considered as an adjunct marker to support clinical risk stratification.

## Background

Acute appendicitis (AA) is one of the leading causes of acute abdominal pain in the emergency room. It has a lifetime prevalence of 7–9% and mainly affects young males. Although its signs and symptoms are common, its diagnosis can be challenging [1–6]. Despite advances in diagnosis and treatment, it has non-negligible morbidity and mortality rates (10% and 1–5%, respectively) [1, 2].

The clinical history and physical examination are the cornerstones in the diagnosis of AA [2, 3]. However, even among experienced surgeons, the diagnostic specificity is approximately 85% [3]. The scores used for AA were developed to improve this diagnosis and have been used routinely to stratify the risk of AA. The best-known scores are the Alvarado, Appendicitis Inflammatory Response (AIR), Raja Isteri Pengiran Anak Saleha Appendicitis (RIPASA), and Adult Appendicitis Scores (AAS) [2, 4, 5]. Although their sensitivity and specificity range between 60 and 90%, none of these scoring systems assess the risk of complications, perforation, or severity [6].

The current consensus does not adequately indicate how to differentiate complicated from uncomplicated AA in the preoperative period. Computed tomography (CT) is the most widely used method for this purpose [2, 7, 8]. However, this examination presents the risk of exposure to ionizing radiation and is unavailable in many emergency departments. The same consensus established that patients with complicated cases should be treated as urgently as possible, while initial cases can be treated with antibiotics alone [2, 3, 8–10]. Therefore, the stratification of this disease into complicated and uncomplicated cases has become increasingly relevant [3, 7, 8].

Based on these limitations, it is imperative to investigate new diagnostic tools, particularly for suspected severe cases. Serum sodium measurement has gained prominence because of its low cost and wide availability. Recent studies have investigated the role of hyponatremia in complicated appendicitis, especially in children [5, 10–13]. Hyponatremia was initially associated with mortality in patients with necrotizing fasciitis. Still, its utility in other diseases, such as gangrenous cholecystitis, intestinal ischemia, colonic perforation, and intestinal fistulae, has recently been established. The pathogenesis of hyponatremia is associated with pro-inflammatory cytokines, such as interleukin-6 (IL-6) and interleukin 1 beta. These interleukins are released during intra-abdominal inflammation, which leads to an increase in circulating vasopressin levels and subsequent sodium dilution [4–6, 9, 14].

The present study aimed to determine whether preoperative serum sodium level may be a predictive marker of severity in patients with acute appendicitis.

## Methods

We retrospectively analyzed 475 patients who underwent appendectomies at the General Hospital of Carapicuíba (Brazil, São Paulo) between January 2018 and February 2023. This study was approved by the Institutional Research Ethics Committee of São Camilo University Center (CAAE number: 68182223.2.0000.0062). The requirement for informed consent was waived due to the retrospective nature of the study. Data were extracted from electronic medical records, including demographic data (age and sex), intraoperative findings, histopathological examinations, and serum sodium levels on admission. Patients who had undergone other concomitant surgeries and those who were not diagnosed with acute appendicitis were excluded. Patients were divided into 2 groups: complicated (n = 254) and uncomplicated (n = 221) AA (Table 1). Cases of necrosis, perforation, regional peritonitis, diffuse peritonitis, and abscesses were considered complicated. A serum sodium level < 136 mEq/l was considered the optimal cutoff point for hyponatremia. Serum sodium levels were measured using the ion-selective electrode method with an Architect Plus C4000 analyzer (Abbott, Lake Bluff, IL, USA).

Categorical variables are presented as percentages and frequencies, and qualitative variables are presented as medians, and interquartile ranges. The Kolmogorov-Smirnov test was used to define normality, whereas the Mann-Whitney U test was used for bivariate comparisons. We used one proportion Z test to analyze the distribution of qualitative factors, analyzing the distribution of relative frequencies (percentages or prevalence). Multivariate logistic regression analyses were performed to evaluate the association of gender, serum sodium level and age with complicated appendicitis. A receiver operating characteristic (ROC) curve was constructed, and sensitivity, specificity, and the area under the curve (AUC) were calculated. Statistical analyses were performed using SPSS version 26 software (IBM Statistics, Armonk, NY, IBM Corp.), and *P* < 0.05 was used as the significance level.

## Results

During the study period, 475 appendectomies were performed, of which 254 (53.5%) were complicated and 221 (46.5%) were uncomplicated (Table 1). AA was more frequent in males (62.3%) (Table 1). The patients had a median age of 22 years and interquartile range (13–34) (Table 1). The median serum sodium level was 137 mEq/L in patients with complicated appendicitis and 139 mEq/L in uncomplicated patients (*P* < 0.001) (Table 2). The serum sodium level showed no difference between both sexes, with females and males having the same median value in complicated cases (137 mEq/L) (Table 2).

**Table 1:** Descriptive analysis of clinical-demographic variables

Characteristic	Complicated	Uncomplicated	*P*-value
**Age**			
Q1-Q3	13–39	13–30	0.127
Median	22.5	21	
**Gender**			
Female	92 (51.4%)	87 (48.6%)	0.541
Male	162 (54.73%)	134 (45.27%)	
**Serum Sodium mEq/L**			
Q1-Q3	135–139	137–140	< 0.001
Median	137	139	
**Hyponatremia** (< 136 mEq/L)	84 (33.1%)	12 (5.4%)	< 0.001
**Acute Appendicitis**			
	254 (53.5%)	221 (46.5%)	0.032

Q1, 1st quartile; Q3, 3rd quartile

**Fig. 1:**
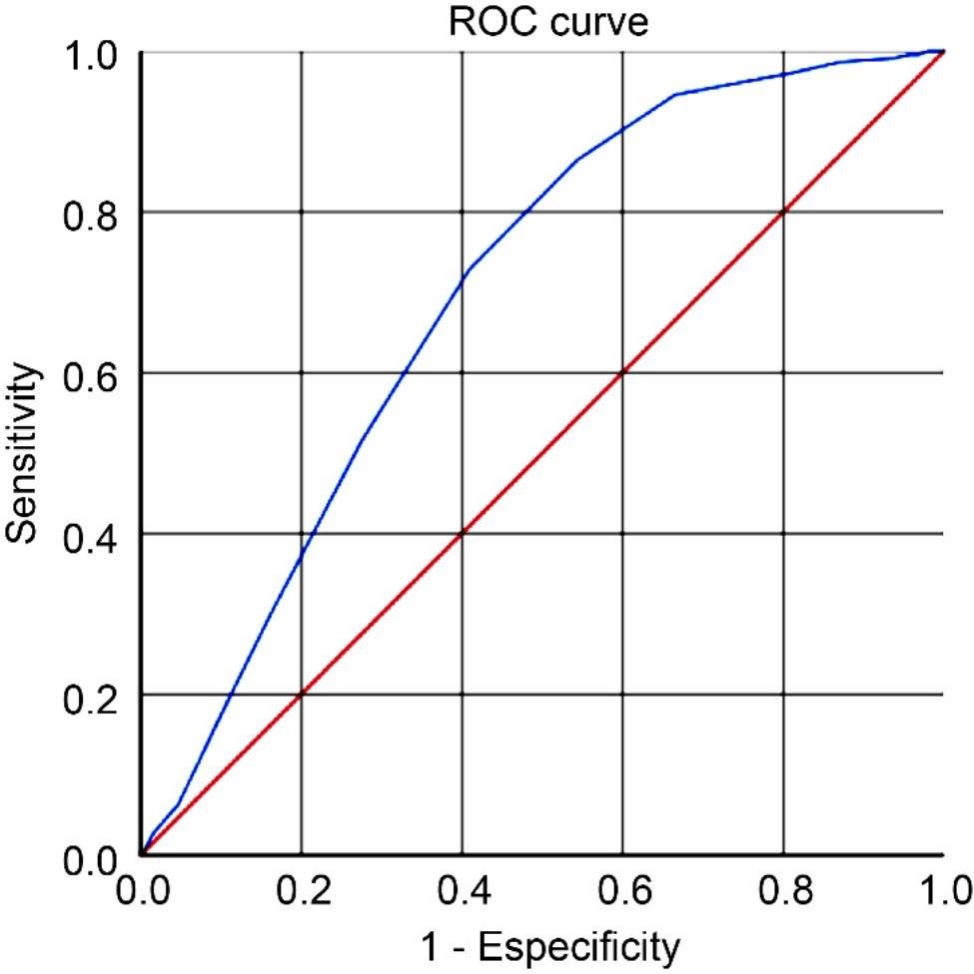
Analysis of the receptor operating characteristic (ROC) curve of serum sodium in patients with complicated acute appendicitis (area under the curve 0.696; *P* < 0.001)

The ROC curve analysis used a serum sodium level of 136 mEq/L as the optimal cutoff point, with a sensitivity of 45.7%, specificity of 86.4%, positive predictive value (PPV) of 79.5%, negative predictive value (NPV) of 58.1%, and AUC of 0.696 (*P* < 0.001) (Fig. 1). Of the 254 patients with complicated appendicitis, 84 (33.1%) had serum sodium levels below 136 mEq/L, whereas only 12 (5.4%) patients with uncomplicated appendicitis had values below this cutoff value.

**Table 2:** Comparison of serum sodium for the type of acute appendicitis

	Female		Male	
	Complicated	Uncomplicated	Complicated	Uncomplicated
Median	137	139	137	138
Q1	135	137,5	135	137
Q3	139	140	139	140
*P*-value	< 0,001		< 0,001	

_Q1, 1st quartile; Q3, 3rd quartile_

The variables (serum sodium, age and gender) were included in a multivariate logistic analysis, which revealed that age (odds ratio: 1,02, 95% CI: 1,01–1,03) and low serum sodium (odds ratio: 0,74, 95% CI: 0,68 − 0,80) levels correlated with the risk of complicated appendicitis. (Table 3). These variables are independent risk factors for complicated appendicitis. Detailed information about multivariate analysis is summarized in Table 3.

**Table 3:** Multivariate logistic model for acute appendicitis

		(B)	Wald	*P*-value	Odds Ratio		
					OR	Lower	Upper
Complicated appendicitis	Serum Sodium	-0,306	48,41	< 0,001	0,74	0,68	0,80
	Age	0,020	9,30	0,002	1,02	1,01	1,03
	Gender (Male)	0,147	0,51	0,475	1,16	0,77	1,73

_(B), Logistic regression coefficient; OR,odds ratio_

## Discussion

Acute appendicitis is one of the main causes of urgent abdominal surgery worldwide [1, 2, 4–6, 15, 16]. In our cohort, lower serum sodium levels were observed in patients with complicated appendicitis, consistent with prior reports. Further prospective studies controlling confounders are required before any predictive value can be established. The limitations of our study include its retrospective design, small sample size, and the fact that data were drawn from a single institution. In addition, there were discrepancies between the hyponatremia cutoff used in our cohort and those adopted in other studies, which reduces the reliability of cross-study comparisons. Another indirect limitation is that negative appendectomies were not included in the analysis because of the small number of such cases. Importantly, several potentially relevant factors that could influence serum sodium were not measured or tested in this article, including patients’ hydration status, dietary intake, body composition (e.g., BMI), baseline renal function, comorbidities, and use of medications that may affect sodium homeostasis. We also lacked standardized information on the timing of sodium measurement in relation to symptom onset and operative management, which may introduce variability in the observed values. Taking together, these constraints limit causal inference and prevent us from asserting serum sodium as an independent predictor of appendicitis severity; rather, sodium should be interpreted as a variable that was associated with severity within this dataset. Although early diagnosis increases the therapeutic success rate, the incidence of perforation can reach up to 40% of cases diagnosed late. Appendiceal perforation is associated with increased morbidity and mortality and mainly affects extremes of age. The evolution of AA into perforation is considered inevitable; however, this dogma is no longer accepted. Recent evidence suggests that the resolution of the condition may be a frequent event [1, 11]. However, acute perforated and non-perforated appendicitis appears to be different disease entities, and perforation occurs more frequently in patients with an impaired inflammatory response [17, 18]. Due to this variability in presentation and evolution, it is pertinent to distinguish preoperatively between complicated and uncomplicated cases [17].

AA can be classified as complicated or uncomplicated based on histopathological findings and classification systems for intraoperative findings [2, 19]. Complicated acute appendicitis occurs when the appendix becomes gangrene or perforates, leading to abscess formation and fecal or purulent peritonitis [4, 5]. The classification by Gomes et al. is a grading system based on intraoperative findings. This system subdivides AA into 5 grades: patients with grades 1 and 2 appendicitis are considered uncomplicated (hyperemia and fibrinous exudate), and those with grades 3, 4, and 5 (3 A, segmental necrosis; 3 B, necrosis of the base of the appendix; 4 A, localized abscess; 4 B, regional peritonitis; and 5, diffuse peritonitis) are considered complicated [19].

Complicated cases are associated with worse clinical outcomes, longer lengths of stay, high hospital costs, poor quality of life, and high complication rates [4, 6, 12, 13]. Early identification of complications seems to have implications for decision-making, especially regarding the appropriate time for surgical intervention. Identifying preoperative diagnostic tools that predict disease severity and aid in decision-making is critical; they may be important in appropriately allocating resources to patients with potentially complicated diseases [14, 15, 20].

Various markers have been used to stratify AA severity. Among these, the most commonly used are C-reactive protein levels (CRP), white blood cell, bilirubin levels, liver inflammatory activity tests (alanine transaminase [ALT] and aspartate aminotransferase [AST]), neutrophil-to-lymphocyte ratio, and serum sodium levels. The study conducted by Akbulut et al. showed that white blood cell > 10.900, total bilirubin > 0.61 and CRP > 0.725 were considered independent risk factors for predicting complicated AA. Farooqui et al. reported that patients with perforated appendicitis had significantly higher levels of white blood cell (*P* = 0.004), bilirubin (*P* < 0.001) and CRP (*P* < 0.001) than patients with a non-perforated appendicitis. A systematic review by Hajibandeh et al. identified that the neutrophil-to-lymphocyte ratio > 8.8 was a predictor of complicated appendicitis (sensitivity of 76.92% and specificity 100% with AUC of 0.91). We identified in our study that serum sodium level < 136 mEq/L has a sensitivity of 45.7% and specificity of 86.4% with AUC: 0.696 to diagnose complicated AA (*P* < 0.001), results very similar to other markers. Recent studies have also associated the use of artificial intelligence and machine learning to predict severity in AA with high levels of accuracy [4–6, 16, 21–23].

Hyponatremia has already been described in other etiologies, such as pneumonia, meningitis, and encephalitis, in which inflammation with or without infection plays a central role. In most diseases, the decrease in serum sodium levels is due to the nonosmotic secretion of vasopressin (ADH). It is becoming increasingly clear that the immunoendocrine interface can release vasopressin directly into circulation. Despite being multifactorial, the current evidence demonstrates the key role of IL-6 in ADH release. The supraoptic and paraventricular nuclei and the subfornical organ are the brain areas involved in this control, translating the osmolarity-control signal. This non-osmotic release of ADH causes reabsorption of excess free water in the kidneys, leading to dilutional hyponatremia [5, 17, 24, 25].

The association between hyponatremia and appendicitis has been investigated more extensively in the pediatric population [26]. The systematic reviews by Gianis et al. and Anand et al. showed similar results regarding the presence of hyponatremia in children with complicated AA. The results were statistically significant in favor of complicated cases. However, only a few papers were included in the reviews, which still shows the paucity of influential articles on the subject [5, 10]. Zhan et al. evaluated children with acute appendicitis and found that patients with complicated diseases had significantly lower serum sodium levels, with a sensitivity of 71%, a specificity of 68%, and an AUC of 0.78.

Pogorolec et al., in their prospective study of 184 children, reported that the mean serum sodium of patients with complicated appendicitis was 132.2 mmol/L and 139.2 mmol/L in patients with uncomplicated appendicitis (*P* < 0.001). A sodium value < 135 mmol/L was used as the best possible cut-off point, with a sensitivity of 94.7%, specificity of 88.5%, AUC of 0.983, and *P* < 0.001 [12]. Meanwhile, in their evaluation of 1283 children under 15 years of age, Walsh et al. considered 135 mEq/L as the cut-off point for hyponatremia and detected hyponatremia in 31.4% of the complicated cases and only 3.8% of the uncomplicated cases (*P* < 0.0001) [27].

Kim et al. evaluated 1550 adult patients who underwent appendectomies. Of the 409 patients with complicated AA, 173 (42.3%) had serum sodium levels of < 135 mEq/L. This same study evaluated that patients with hyponatremia are almost 3 times more likely to have perforated or gangrenous appendicitis [20]. Our study identified that patients with hyponatremia were 5 times more likely to have complicated appendicitis. (odds ratio [OR]: 5.35; 95% confidence interval [CI]: 3.39–8.45). In a study by Turhan et al. evaluating more than 700 patients, including adults and children, 137.5 mEq/l was used as the cut-off point. This value had a sensitivity of 63%, a specificity of 66%, and an AUC of 0.67. (*P* < 0.0001) [14].

The study conducted by Symeonidis et al. in adult patients showed a sensitivity of 41.4% and specificity of 98.3% with a cutoff value of < 135 mEq/L for serum sodium. This study identified a PPV of 96.6% and an NPV of 60%. Hyponatremia was found in 41.2% of complicated patients and 1.6% of uncomplicated patients (*P* < 0.001) [26]. Our study identified a sensitivity of 45.7% and specificity of 86.4% with a cut-off value of 136 mEq/L (AUC: 0.696, *P* < 0.001). Hyponatremia was found in 33.1% of the patients with complicated AA, data very similar to those found by Pogorolec et al. and Kim et al. (31.4% and 42.3%, respectively).

A retrospective study by Sheen et al. on adult and minor patients identified significant findings only in the adult population. The authors reported that the risk in a patient with hyponatremia and complicated appendicitis was almost eight times higher. (OR: 7.915 95% CI: 2.7656–22.6521); *P* < 0.0001 [17]. Ozdemir et al. retrospectively analyzed 772 adult patients who underwent an emergency appendectomy. A sensitivity of 27.8% and specificity of 92.1% were identified with a cutoff value of 133.5 mEq/L and an AUC of 0.612 (95% CI: 0.539–0.684). The same authors found that patients with hyponatremia (sodium ≤ 134 mEq/L) were 3 times more likely to have complicated AA [28].

Clinical trials, systematic reviews, and consensus statements (World Society of Emergency Surgery and American Association for the Surgery of Trauma) include non-surgical treatment of AA in their recommendations [2, 7, 8, 29]. CODA (Comparison of Appendicectomy and Antibiotic Drugs) and APPAC TRIAL (Antibiotic Therapy vs. Appendicectomy for Treatment of Uncomplicated Acute Appendicitis) are relevant recent clinical trials on the non-surgical treatment of AA. These trials used computed tomography (CT) to select candidate patients for nonsurgical treatment [7, 8]. Studies have shown that patients selected by imaging exams for nonsurgical treatment may have an early failure (24–72 h) rate of 8–12% [29]. A recent meta-analysis of more than 4000 patients evaluated for complicated CT findings (extraluminal appendicitis, abscess, appendiceal wall defect, extraluminal air, ileus, perpendicular fluid collection, ascites, and intraluminal air) showed a pooled specificity greater than 70% (70–100%). However, the sensitivity was quite limited, ranging from 14 to 59% [30]. Another point worth mentioning is the lack of availability of imaging tests (CT) for diagnosing complicated AA in many emergency services and their high cost for countries with limited resources. Identifying a strong predictor of appendiceal perforation that is inexpensive and widely available, such as sodium, could guide the indication for the use of imaging methods and help in the selection of patients with radiologically confirmed AA who would be the best candidates for nonsurgical treatment [12, 25, 31]. The association of other serological markers, such as the neutrophil/lymphocyte ratio, increases the diagnostic and predictive value and could increase assertiveness in complicated cases [3–6, 32].

Few studies have investigated which parameters should be used to select the patients with the best chance of success with non-surgical treatment [29]. In the retrospective study by Loftus et al. with 81 patients, an appendix diameter < 13 mm was associated with a higher probability of success with conservative treatment [33]. In a randomized clinical trial, patients without appendicolith responded better to antibiotic therapy than those with appendicolith (41 vs. 25%, *P* < 0.05) [7]. However, another study evaluated the sensitivity and PPV to identify appendicolith on preoperative computed tomography. A sensitivity of 53.1% and PPV of 44.8% were identified, showing that tomography is not a reliable method for identifying appendicolith preoperatively [34]. Singh et al. evaluated 1014 appendectomies and identified that appendicolith was an incidental finding, and its prevalence was too low to be considered a cause of appendicitis. Appendicoliths were found in 18.1% of confirmed appendicitis cases and 28.6% of negative appendicitis cases. Chandrasegaram et al. evaluated more than 4000 patients who underwent appendectomy and identified the presence of appendicolith in only 3.9% of cases, and in the vast majority (60.5%), appendicolith was found in normal appendices [35]. Obstruction of the appendiceal lumen by a fecalith has been thought to lead to distension, bacterial growth, increased pressure, and progressive involvement with gangrene and appendiceal perforation [18]. Arnbjörnsson et al. evaluated the intraluminal pressure of 33 patients with AA. These authors identified an increase in luminal pressure in only 25% of these patients [36]. Flum et al. reported that appendiceal perforation is more associated with an altered inflammatory response than with an increase in intraluminal pressure due to obstruction [5, 18]. More than one mechanism is thought to be responsible for causing AA (obstruction, inflammatory response, microbiome, trauma, diet, constipation, and infection) [34]. Therefore, the use of markers such as sodium released by the inflammatory stimulus (IL-6) could help select patients for non-surgical treatment. These observations will help establish new selection criteria for patients who are candidates for non-surgical treatment of AA in future clinical trials.

In addition to being a predictive marker of severity in patients with AA, the preoperative measurement of serum sodium can potentially affect the management of patients who are candidates for conservative treatment and optimize decision-making in complicated cases [12, 26, 28].

The limitations of our study include its retrospective design, small sample size, data from a single institution, and discrepancies with the cutoff values used for hyponatremia in other studies, making a comparison between studies less reliable. An indirect limitation was not including negative appendectomies in the analysis due your small number of cases. Large-scale multicenter prospective studies are required to confirm our findings and extend them to clinical practice.

## Conclusion

Thus, hyponatremia appears to be a promising marker for predicting the severity of acute appendicitis. The measurement of serum sodium levels  < 136 mEq/L is an additional tool and may be indicative of complicated appendicitis. Due to its low cost and wide availability, it has become an extremely relevant marker.

## Data Availability

The datasets used and/or analysed during the current study available from the corresponding author on reasonable request.

## Abbreviations

AA: Acute appendicitis
AAS: Acute appendicitis score
ADH: Vasopressin
AIR: Appendicitis inflammatory response
ALT: Alanine transaminase
AST: Aspartate aminotransferase
AUC: Area under the curve
(B): Logistic regression coefficient
CI: Confidence interval
CODA: Comparison of appendectomy and antibiotic drugs
CRP: C-reactive protein
CT: Computed tomography
SD: Standard deviation
IL-6: Interleukin-6
NPV: Negative predictive value
OR: Odds ratio
PPV: Positive predictive value
Q1: First quartile
Q3: Third quartile
RIPASA: Raja Isteri Pengiran Anak Saleha Appendicitis
ROC: Receiver operating characteristic
