# Correction: Serum sodium as an adjunct marker of acute appendici­tis severity: a retrospective cohort study

**DOI:** 10.1186/s12893-026-03927-8

**Published:** 2026-06-15

**Authors:** Bruno Messias, Isabella Cubas, Caio Oliveira, Flavia Hashimoto, Erica Mocchetti, Tania Ichinose, Jaques Waisberg, Marcelo A. F Ribeiro Junior

**Affiliations:** 1 Department of Surgery, General Hospital of Carapicuiba, 5, Pedreira street, Carapicuiba, SP 06321-665 Brazil; 2 Medical School, São Camilo University Center, Nazare Avenue, 1501, São Paulo, SP 04263- 200 Brazil; 3 Department of Surgery, ABC Medical School, Lauro Gomes Avenue, Santo André 2000, SP 09060-870 Brazil; 4 Critical Care and Acute Care Surgery, Sheikh Shakhbout Medical CityMayo Clinic, P. O. Box 11001, Abu Dhabi, United Arab Emirates; 5 Catholic University of São Paulo, 290, Joubert Wey Street, Sorocaba, SP 18030-070 Brazil

**Correction: BMC Surg 23, 312 (2023)**

**https://doi.org/10.1186/s12893-023-02224-y**

In article [1], After publication, and following post-publication peer review by the Journal, several statements in this Article were considered to overstate the possible usefulness of serum sodium levels as a marker of acute appendicitis severity. The authors are therefore making the following corrections to the Article:

The title has been changed from “Usefulness of serum sodium levels as a novel marker for predicting acute appendicitis severity: a retrospective cohort study” to “Serum sodium as an adjunct marker of acute appendicitis severity: a retrospective cohort study”;

Abstract, paragraph 4. The Conclusions sub-section has been changed from “Preoperative serum sodium levels are a useful tool for predicting the severity of acute appendicitis. Due to its low cost and wide availability, it has become an extremely relevant marker” to “Preoperative serum sodium levels may help in assessing the severity of acute appendicitis. Given their low cost and wide availability, sodium measurements could be considered as an adjunct marker to support clinical risk stratification”;

Discussion section, paragraph 13. The following text was added after sentence 1: “In our cohort, lower serum sodium levels were observed in patients with complicated appendicitis, consistent with prior reports. Further prospective studies controlling confounders are required before any predictive value can be established. The limitations of our study include its retrospective design, small sample size, and the fact that data were drawn from a single institution. In addition, there were discrepancies between the hyponatremia cutoff used in our cohort and those adopted in other studies, which reduces the reliability of cross-study comparisons. Another indirect limitation is that negative appendectomies were not included in the analysis because of the small number of such cases. Importantly, several potentially relevant factors that could influence serum sodium were not measured or tested in this article, including patients’ hydration status, dietary intake, body composition (e.g., BMI), baseline renal function, comorbidities, and use of medications that may affect sodium homeostasis. We also lacked standardized information on the timing of sodium measurement in relation to symptom onset and operative management, which may introduce variability in the observed values. Taking together, these constraints limit causal inference and prevent us from asserting serum sodium as an independent predictor of appendicitis severity; rather, sodium should be interpreted as a variable that was associated with severity within this dataset.”

Conclusion section, paragraph 1: the text was changed from “Thus, hyponatremia appears to be a promising marker for predicting the severity of acute appendicitis. The measurement of serum sodium levels < 136 mEq/L is an additional tool and may be indicative of complicated appendicitis. Due to its low cost and wide availability, it has become an extremely relevant marker.” to “Thus, hyponatremia appears to be a promising marker for predicting the severity of acute appendicitis. The measurement of serum sodium levels < 136 mEq/L is an additional tool and may be indicative of complicated appendicitis. Due to its low cost and wide availability, it has become an extremely relevant marker.

The original article has been corrected.
